# Paving the Way for Immunotherapy in Pediatric Acute Myeloid Leukemia: Current Knowledge and the Way Forward

**DOI:** 10.3390/cancers13174364

**Published:** 2021-08-28

**Authors:** Joost B. Koedijk, Inge van der Werf, Friso G. Calkoen, Stefan Nierkens, Gertjan J. L. Kaspers, Christian Michel Zwaan, Olaf Heidenreich

**Affiliations:** 1 Princess Máxima Center for Pediatric Oncology, 3584 CS Utrecht, The Netherlands; i.m.vanderwerf@prinsesmaximacentrum.nl (I.v.d.W.); f.g.j.calkoen-2@prinsesmaximacentrum.nl (F.G.C.); s.nierkens-2@prinsesmaximacentrum.nl (S.N.); g.j.l.kaspers@prinsesmaximacentrum.nl (G.J.L.K.); c.m.zwaan@prinsesmaximacentrum.nl (C.M.Z.); 2 Center for Translational Immunology, University Medical Center Utrecht, 3584 CX Utrecht, The Netherlands; 3 Department of Pediatric Oncology, Emma Children’s Hospital, Amsterdam UMC, Vrije Universiteit, 1105 AZ Amsterdam, The Netherlands; 4 Department of Pediatric Oncology, Erasmus MC/Sophia Children’s Hospital, 3015 GD Rotterdam, The Netherlands; 5 Wolfson Childhood Cancer Research Centre, Newcastle University, Newcastle upon Tyne NE1 7RU, UK

**Keywords:** acute myeloid leukemia, immune landscape, tumor immune microenvironment, immune profiling, immune monitoring, immunotherapy, children

## Abstract

**Simple Summary:**

Immunotherapy may be an attractive treatment option to increase survival, and to reduce treatment-related side effects, for children with acute myeloid leukemia (AML). While immunotherapies have shown successes in many cancer types, the development and subsequent clinical implementation have proven difficult in pediatric AML. To expedite the development of immunotherapy, it will be crucial to understand which pediatric AML patients are likely to respond to immunotherapies. Emerging research in solid malignancies has shown that the number and phenotype of immune cells in the tumor microenvironment is predictive of response to several types of immunotherapies. Such a predictive model may also be applicable for AML and, thus, knowledge on the immune cells infiltrating the bone marrow environment is needed. Here, we discuss the current state of knowledge on these infiltrating immune cells in pediatric AML, as well as ongoing immunotherapy trials, and provide suggestions concerning the way forward.

**Abstract:**

Immunotherapeutic agents may be an attractive option to further improve outcomes and to reduce treatment-related toxicity for pediatric AML. While improvements in outcome have been observed with immunotherapy in many cancer types, immunotherapy development and implementation into patient care for both adult and pediatric AML has been hampered by an incomplete understanding of the bone marrow environment and a paucity of tumor-specific antigens. Since only a minority of patients respond in most immunotherapy trials across different cancer types, it will be crucial to understand which children with AML are likely to respond to or may benefit from immunotherapies. Immune cell profiling efforts hold promise to answer this question, as illustrated by the development of predictive scores in solid cancers. Such information on the number and phenotype of immune cells during current treatment regimens will be pivotal to generate hypotheses on how and when to intervene with immunotherapy in pediatric AML. In this review, we discuss the current understanding of the number and phenotype of immune cells in the bone marrow in pediatric AML, ongoing immunotherapy trials and how comprehensive immune profiling efforts may pave the way for successful clinical trials (and, ultimately, implementation into patient care).

## 1. Introduction

Acute myeloid leukemia (AML) is a heterogeneous blood cancer characterized by both aberrant proliferation and arrested differentiation of immature myeloid cells in the bone marrow [1]. Due to intensified chemotherapy, risk-adapted treatment, improvement in allogeneic stem cell transplantation (allo-SCT) and optimized supportive care, survival of pediatric AML has greatly improved over the last decades [2]. Nevertheless, 20–30% of children with AML do not survive as a result of significant treatment-related toxicity and death due to relapse [3]. Furthermore, survivors often experience serious side effects and late effects due to the treatment [3]. Therefore, alternative treatment options that further improve outcome and reduce treatment-related side effects are required.

To date, therapeutic options that make use of T-cell-mediated effects to eliminate residual leukemic cells, such as allo-SCT, have shown to evoke anti-AML immunity and support the use of immunotherapy in pediatric AML [4,5,6,7]. However, allo-SCT is associated with major side effects such as chemotherapy- or irradiation-related toxicities and graft-versus-host-disease [8]. Hence, less toxic immunotherapy options that enhance anti-leukemic immune surveillance without these long-term sequelae are highly needed for this disease. Encouraged by the initial successes of immunotherapy in acute lymphoblastic leukemia (ALL) and various solid cancers, relatively new immunotherapeutic options are now available or under development for AML: immune checkpoint inhibitors (ICIs), unconjugated and bispecific antibodies, adoptive cell therapy, cytokines and other immune-modulating soluble factors, vaccines, and oncolytic viruses (Table 1) [6,7,9]. Antibody-drug conjugates such as gemtuzumab-ozogamicin are not considered as immunotherapy in this overview, as their main action is targeted delivery of cytotoxic agents to tumor cells.

Unfortunately, clinical translation from basic immunobiology to effective immunotherapy appears to be relatively slow for both adult and pediatric AML in comparison to other cancer types [4]. For instance, blinatumomab, a bispecific antibody binding CD19 and CD3, and CD19-directed chimeric antigen receptor (CAR) T-cells, have been approved for clinical use in both adults and children with B-cell precursor ALL [13,14,15]. Furthermore, ICIs have revolutionized the treatment of advanced melanoma and Hodgkin’s disease as well as other cancer types [16,17]. In contrast, none of these relatively new immunotherapies have been approved for clinical use for AML due to disappointing results in clinical trials [18,19,20,21].

The results in these trials and, consequently, the relatively slow implementation of immunotherapy in patient care can be explained in part by an incomplete understanding of the microenvironment in which the leukemic cells reside. For AML, the alleged tumor microenvironment (TME) is the bone marrow and includes, next to tumor cells, immune and other normal hematopoietic, stromal, and endothelial cells [22]. During the leukemic transition, tumor cells grow at the expanse of normal hematopoietic cells, successfully evade or suppress immune surveillance, and consequently create a bone marrow microenvironment that is immunologically dysfunctional [6]. Emerging research in solid malignancies has shown that the number and phenotype of immune cells in the TME is predictive of response to several types of immunotherapies [23,24,25,26,27,28]. For adult AML, a similar trend is observed in early phase clinical trials [29,30,31]. However, the abundance and phenotype of pro- and anti-tumorigenic immune cells in the TME in adult and pediatric AML is hardly explored. In addition, the development of antigen-directed immunotherapies such as CAR T-cells has been hampered by a lack of tumor-specific antigens in AML [4]. Most tumor-associated antigens, such as CD33 and CD123, overlap with antigens expressed on normal hematopoietic progenitor cells [4,5,6]. Targeting those antigens with CAR T-cells may result in profound myelosuppression due to lack of specificity and, hence, may mainly be a bridge to transplant [4,5,6]. In addition, immunotherapies have been associated with severe off-target systemic toxicities, including cytokine-release syndrome, immune cell-associated neurotoxicity syndrome, and graft-versus-host-disease [32].

Extensive characterization of immune cells in the TME is crucial to tackle these challenges and to design successful immunotherapy trials [3,4,5,6,7,8,9,10,11,12,13,14,15,16,17,18,19,20,21,22,23,24,25,26,27,28,29,30,31,32,33,34,35,36,37]. Such characterization efforts may capture the evolution of the bone marrow microenvironment during therapy and disease progression, and consequently reveal which treatment time points are suitable for immunotherapeutic intervention. Moreover, tumor-associated antigen detection on AML blasts must be part of these efforts to support the use of antigen-directed immunotherapies. Importantly, characterization efforts that focus on the pediatric population are necessary as insights gained from adult AML patients might not be directly applicable to children with AML. This may be due to differences in underlying leukemia biology, treatment, and potentially in host-factors such as the immune cell composition in the bone marrow [29,38]. In this review, we describe the current knowledge on both immune cells and target antigen expression in the TME in pediatric AML, discuss ongoing immunotherapy trials, and delineate how immune profiling efforts may pave the way for immunotherapy implementation into patient care.

## 2. Immune Cells in the TME

**Figure 1:**
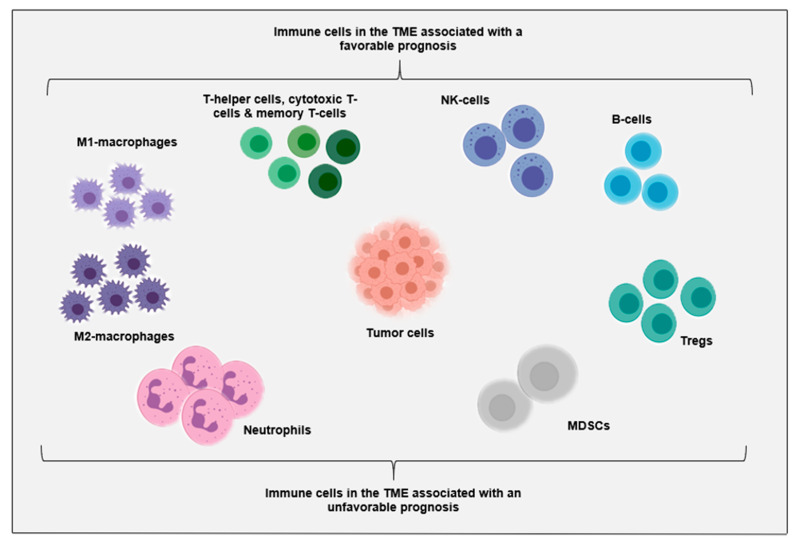
Overview of prognostically relevant immune cell populations in the tumor microenvironment as described in solid malignancies. MDSCs: myeloid-derived suppressor cells; NK: natural killer; Tregs: regulatory T-cells. For a detailed overview of relevant receptors, ligands and expression states of these immune cell populations in AML, we refer the reader to two excellent reviews [4,5].

Immune cells and immune-related factors such as soluble molecules play a major role in cancer development and progression [4]. For example, T- and natural killer (NK)-cells are considered essential for effective anti-tumor immunity, while M2-macrophages and myeloid-derived suppressor cells exert tumor-supporting activities in the TME [22,23,24]. The importance of the immune system can be exemplified by the prognostic and predictive value of both the number and phenotype of immune cells in the TME for both immunotherapy interventions and conventional anticancer therapies in many cancers [22,23,24,25,26,27,28]. As a result of enormous immune profiling efforts in solid malignancies, a general overview of critical players in the TME has been generated (Figure 1). Accordingly, cytotoxic T-cells, memory T-cells, T-helper 1 cells, follicular helper T-cells, NK-cells, B-cells, and M1-macrophages have been associated with prolonged survival, while high densities of regulatory T-cells (Tregs), M2-macrophages, myeloid-derived suppressor cells, and neutrophils have been correlated with poor prognosis [23,39,40,41]. Although the prognostic significance of most immune cell types in AML remains to be elucidated, a similar trend has been seen in recent studies on adult AML [42,43,44]. For instance, for adult AML patients treated with chemotherapy, a relatively high proportion of T- and NK-cells in the bone marrow at diagnosis has been associated with improved survival, while the proportion of Tregs has been associated with poor prognosis [42,43,44]. For pediatric AML, such data are not available in literature yet. Since the prognostic importance of immune cells in the TME is increasingly recognized, further improvement of our understanding of immune cells in both adult and pediatric AML will be necessary to move towards immunological-driven biological approaches.

As only a minority of patients respond in most immunotherapy trials, many studies have focused on elucidating factors that impact the response to immunotherapy. For instance, studies in both solid and hematological cancers have shown that patients with an immune-infiltrated or ‘hot’ TME have a relatively high probability of response to ICIs [23,24,25,26,27]. Such an environment has been characterized by high abundance of both CD4+ and CD8+ T-cells, opposed to low T-cell abundance in an immune-depleted or ‘cold’ TME. Interestingly, this observation has not only been seen for ICIs, but also for other immunotherapeutic options such as bispecific antibodies, adoptive cell therapy and vaccines [26,27,29,30]. Moreover, beneficial proportions of CD4+ and CD8+ T-cell phenotypes, such as an increased ratio of memory-like CD8+ T-cells compared to exhausted CD8+ T-cells, have appeared to be another factor that impacts immunotherapy response [28].

Immunotherapy studies in adult AML have shown similar observations [26,27,28]. For example, a phase II study that evaluated nivolumab (anti-PD1 ICI) in combination with the hypomethylating agent azacytidine in 70 adults with relapsed or refractory AML showed that patients with a pre-treatment bone marrow T-cell proportion ≥ 13.2%, using flow cytometry, had a 74% likelihood of response [31]. In addition, another phase II study assessed the antileukemic activity of the bispecific antibody flotetuzumab in 30 adults with refractory or relapsed AML [29,30]. This study did not use quantitative measurements of T- and other immune cells, but instead estimated the abundance of immune cells in the bone marrow based on RNA-sequencing data. The results showed that out of the 17 patients with an immune-infiltrated subtype, 11 patients showed anti-leukemic activity. In contrast, among the 13 patients with an immune-depleted subtype, only one patient responded [29,30]. Unfortunately, most immunotherapy trials in adult AML did not measure T-cell and other immune-cell levels before treatment to date. To improve our understanding of which patients with AML are likely to respond to certain immunotherapies, immune-based biomarkers need to be identified and tested in prospective clinical trials. For pediatric AML, no studies on the predictive value of immune cells have been completed.

### 2.1. Immune Cells in the TME of AML at Diagnosis

Since the prognostic and predictive potential of immune cells in the bone marrow of AML is increasingly recognized, we summarized the available information on immune cell abundance and phenotype in the TME for children with AML at diagnosis. To date, few studies have examined these parameters on a protein level. However, recent microarray- and RNA-sequencing based studies have provided valuable information [29,45]. These studies employed gene scores that either reflected the abundance of multiple immune cell types or focused specifically on T- and NK-cell abundance. An extensive study by Dufva and colleagues that used a 5-gene “cytolytic” score specific for T- and NK-cell abundance showed that children with AML (*n* = 273) had a median combined proportion of T- and NK-cells of nearly 25% out of all living cells in the bone marrow at diagnosis [45]. Of interest, the T- and NK-cell abundance in children did not differ from adults with AML (*n* = 1585) in the same study [45]. Similar results were observed in a study with 34 children and 334 adults with AML that used RNA-sequencing to estimate the abundance of multiple immune cells including antigen-presenting cells, T, B-, and NK-cells [29]. Only adults with AML ≥ 60 years (*n* = 102) showed a slightly higher immune cell abundance in comparison to children with AML [29]. This may be related to the increased prevalence of myelodysplastic syndrome-related AML in older adults as this subtype is associated with relatively high levels of immune infiltration in the bone marrow [45,46].

In contrast, a small study that used flow cytometry in children with AML (*n* = 28) revealed a significantly lower fraction of T-cells out of all mononuclear cells in the bone marrow (4%) [44]. Phenotypically, both CD4+ and CD8+ T-cells expressed higher levels of the inhibitory checkpoints LAG3 and PD-1, in comparison to healthy donors [47]. These checkpoints have been associated with exhaustion and reduced functional capacity of T-cells [48]. However, T-cells with expression of LAG3 and PD-1 still retained modest capacity for cytokine production, indicating that they were not fully exhausted [47]. Comparably, despite significant declines in granzyme, CD16, CD57, and NKG2D, NK-cells in children with AML still showed functional activity [47].

In addition, clonotype diversity of T- and BCRs can inform whether patients can generate an effective antigen-specific anti-tumor immune response, for instance after immunotherapy [48]. High levels of T- and B-cell clonal expansion indicate activation of these cells and have been associated with improved responses to immunotherapy [49]. However, a growing body of evidence suggests that many T-cells in the TME are simply ‘observers’ that are incapable of recognizing and eliminating tumor cells and accordingly present low levels of clonal expansion [50]. In a study focused on T-cell receptor (TCR) and B-cell receptor (BCR) analysis of diagnostic bone marrow transcriptomes in both adult (*n* = 151) and pediatric AML (including infant AML, up to 1 year of age; *n* = 145 in total), T-cells in infant AML presented relatively low levels of clonal expansion in comparison to pediatric and adult AML [51]. It was suggested that this might be due to limited bacterial and viral antigen exposure prior to therapy. Notably, there were no differences in clonal expansion between pediatric and adult AML. However, adult AML samples in this study had significantly more secondary immunoglobulin class switch events than pediatric AML samples. These results indicate higher levels of B-cell activation in adult AML in comparison to pediatric AML.

Taken together, microarray- and RNA-sequencing based studies have provided valuable insights into the immune cell abundance in the bone marrow of children with AML. Apart from adults with AML ≥ 60 years, children with AML appeared to have similar levels of T- and NK-cell abundance in the bone marrow at diagnosis in comparison to adults with AML. Moreover, the high proportions of T- and NK-cells in the bone marrow of children with AML in these studies look promising and anticipate that children with AML may benefit from immunotherapy in the future. Studies that evaluated the immune cell abundance and phenotype on the protein level at diagnosis are scarce in children with AML. Although the microarray- and RNA-sequencing based methods in the discussed studies have been validated and were found to robustly estimate immune cell levels as measured by protein-based assays such as flow cytometry, functional studies are needed to deepen our understanding of the phenotype of immune cells in the bone marrow in children with AML.

### 2.2. Immune Cells in the TME in Relapsed and Refractory Disease

Unfortunately, the abundance and phenotype of immune cells in the TME of relapsed and refractory pediatric AML has not been elucidated yet. In addition, no trials on immunotherapy drugs for relapsed and refractory pediatric AML have been published to date. For adults, most data on the immune cell abundance in relapsed and refractory AML stem from the studies that measured these parameters before treatment in immunotherapy trials [29,30,31]. For instance, the study that evaluated nivolumab in combination with azacytidine in relapsed and refractory adult AML showed that responders had a higher frequency of pretherapy T-cells out of all living cells in the bone marrow as measured by flow cytometry in comparison to non-responders (32.5% vs. 17.5%) [31]. Furthermore, data on the phenotype of immune cells in the TME in the relapse setting suggested that cytotoxic T-cells fail to restrain leukemia growth [29]. For instance, one study that employed RNA-sequencing reported that in comparison to the diagnostic setting, cytotoxic T-cells showed increased markers of terminal differentiation, senescence, and exhaustion at relapse [29]. Data from another study confirmed that cytotoxic T-cells showed upregulation of exhaustion markers at relapse in comparison to healthy donors, but this was also seen at diagnosis [52]. Moreover, cytotoxic T-cells in adult AML showed wide signs of impairment and exhaustion at relapse after allo-SCT [53]. Since most immunotherapy trials for AML test immunotherapeutic strategies in relapsed and refractory patients, it is key to unravel these parameters to improve the use of immunotherapies in future clinical trials.

### 2.3. Immune Cells in the TME during and after Therapy

Over the years, the use of immunotherapy during and after conventional therapy has gained interest in the field of AML due to the relatively low leukemic burden in these settings [54]. Accordingly, relatively low numbers of blasts in the bone marrow might lead to a reduction in immunosuppressive signals and more space and nutrition for immune cells [55]. Indeed, one study revealed that the RNA-sequencing based estimates of immune cell abundance in the adult AML TME were inversely associated with the leukemic burden at diagnosis [29]. Furthermore, the RNA-sequencing based estimated immune cell abundance was significantly higher in adult AML patients in complete remission versus diagnosis [29]. Therefore, special attention has been dedicated to the potential use of immunotherapy for the eradication of minimal residual disease (MRD) in AML and other hematological cancers. For instance, in adults with B-cell precursor ALL, out of 21 MRD-positive patients, MRD conversion from positive to negative was achieved in 80% of patients after one cycle of treatment with the bispecific antibody blinatumomab [56]. Consequently, blinatumomab has been approved for the treatment of MRD in B-cell precursor ALL [56,57]. For AML, this study in combination with several other preclinical studies have added interest in the eradication of MRD with immunotherapy [58,59]. For instance, in a mouse model of AML with MRD-positivity, blocking of the immune checkpoint axis with ICIs resulted in prolonged survival in comparison to no treatment [58]. Furthermore, preliminary results from an ongoing phase II study that evaluates the anti-PD1 ICI nivolumab in adult AML patients in complete remission, showed encouraging results [59]. In particular, 71% of patients were in continuing complete remission at 12 months after treatment (ClinicalTrials.gov Identifier: NCT02532231) despite their high risk of relapse as indicated by persistent MRD or adverse prognostic factors [59].

To date, few clinical trials have assessed the role of immunotherapy as maintenance therapy for children with AML [60,61,62]. The randomized ELAM02 phase III trial evaluated whether the use of interleukin-2 after consolidation therapy would improve disease-free survival for newly diagnosed children with AML. Unfortunately, no differences in disease-free and overall survival were observed between the intervention and control arm [60]. Similarly, two phase II clinical trials reported no improvements in disease-free and overall survival with expanded NK-cell infusions after consolidation therapy for newly diagnosed pediatric AML [61,62]. Although these initial trials did not show a clinical benefit of immunotherapeutic maintenance therapy in pediatric AML, this does not preclude the potential usefulness of (combinations of) recently developed immunotherapeutic agents as consolidation therapies. Furthermore, changes in immune cell abundance and phenotype in the TME during or after therapy in pediatric AML have not been studied. Therefore, it is currently unknown how current treatment protocols affect the TME in pediatric AML and thus, which treatment time points are particularly suitable for immunotherapy intervention.

For adult AML, these data are also scarce. As mentioned above, one study revealed that the RNA-sequencing based immune cell abundance was significantly higher in complete remission in comparison to diagnosis in adults with AML (*n* = 22) [29]. Furthermore, CTLA4 expression was upregulated, while CD244 coinhibitory molecule was downregulated, which suggests T-cell activation after induction therapy [29]. Another study in a larger adult cohort (*n* = 72) reported equal results in terms of T-cell activation after induction therapy in responders, while non-responders showed relatively high levels of dysfunction in comparison to the pretreatment setting [63]. However, while some chemotherapeutic agents may indeed activate antitumor immune responses, these agents might concomitantly induce tolerogenic and immunosuppressive pathways [64]. For example, early lymphocyte recovery in 20 adult patients undergoing induction chemotherapy for newly diagnosed AML indicated that recovering T-cells in the peripheral blood were predominantly activated Tregs with suppressive activity [65,66,67].

Collectively, the use of immunotherapy during and/or after conventional treatment has the potential to support the eradication of MRD and consequently prevent relapsed disease. However, data on the immune cell abundance and phenotype is limited for both adult and pediatric AML. Since studies across a spectrum of cancers have observed plasticity of immune cell numbers and their phenotypes before- and after conventional treatment, delineating changes in bone marrow immune cell abundance and phenotype for pediatric AML will likely be important for the selection of suitable treatment time points for immunotherapy intervention [68,69,70,71,72].

### 2.4. Genetic Alterations That Affect the Immune Cell Abundance in the TME

Next to the fact that leukemic cells compete with immune cells for the bone marrow niche occupancy, emerging research suggests that certain genetic alterations in the AML cells may as well impact the immune cell abundance in the TME [45,73,74]. For instance, the earlier mentioned microarray- and RNA-sequencing based study by Dufva and colleagues that included both pediatric and adult AML patients reported a strong positive correlation between the cytolytic score and TP53 mutations, and between the cytolytic score and a myelodysplastic syndrome-like signature [45]. Conversely, common adult AML driver mutations FLT3 and NPM1 were associated with a lower cytolytic score. These findings present important associations between molecular alterations and immune infiltration in the TME. Although the interplay between genetic alterations and the immune landscape is still poorly elucidated in AML, future research on this topic may be able to improve risk stratification and consequently broaden therapeutic approaches for specific subgroups of patients [73]. Furthermore, since pediatric AML differs from adult AML in being mainly a fusion-driven disease, this may require separate pediatric studies [38,75].

## 3. The Relevance of Systemic Immunity for Immunotherapy Responses

Over the last decade, the field of immuno-oncology has focused heavily on understanding the immune cells in the TME. However, the immune system is coordinated across tissues and communication with the peripheral blood (PB) is essential for immune function in the TME [76]. Recently, emerging evidence suggested that intact peripheral immune function, communication, and trafficking are required for ICI efficacy. Mechanistically, several reports suggested that ICIs drive novel, non-exhausted T-cell clones into the TME that were not present locally prior to therapy [77,78,79].

In pediatric AML, only one study that used flow cytometry examined the presence of immune cells in the PB [80]. This study examined the abundance of Tregs in the PB of 25 children with AML in serial assessments—diagnosis, post-induction, post-consolidation, three and six-months follow-up and relapse. The authors reported that Treg levels were increased at diagnosis in comparison to healthy donors. Tregs significantly decreased after induction chemotherapy and continued to decrease over the course of treatment and during follow-up for patients that remained in complete remission. In patients who relapsed, Treg levels remained constant from the post-induction time point up to the last follow-up preceding relapse, and then increased at relapse presentation again. Although this study solely focused on Tregs and did not capture the range of other immune cells in the PB, it suggests a role for Tregs in disease progression of AML. These results are in line with observations in adult AML, where a relatively high proportion of Tregs in the bone marrow at diagnosis was associated with inferior survival [43].

Studies that have assessed the immune reconstitution in the PB after allo-SCT might also be informative of whether immunotherapy is likely to be effective in this setting. For instance, T-cell reconstitution was shown to be excellent within one to two months after unrelated cord blood donor allo-SCT in pediatric AML in the absence of anti-thymocyte globulin as part of the conditioning regimen [81,82,83,84]. When serotherapy was part of the conditioning regimen without dose optimization for each individual patient, it negatively impacted immune reconstitution [83,84]. While the addition of serotherapy reduced graft rejection and graft-versus-host-disease, it increased the incidence of viral infections and leukemia relapses [82]. Therefore, the effects of the conditioning regimen should be considered when immunotherapy is used to augment the graft-versus-leukemia effect. Furthermore, as illustrated in the review by Soiffer et al., the use of immunotherapy after allo-SCT might lead to uncontrollable toxicity, such as graft-versus-host-disease [85]. For example, a phase I study that used the anti-PD1 ICI nivolumab as maintenance therapy after allo-SCT in adult patients with evidence of MRD (ClinicalTrials.gov Identifier: NCT02985554) was stopped prematurely due to an unexpected number of high-grade immune-related adverse events [86].

Taken together, the research community has only just begun to recognize the role of immune cells in the PB for achieving responses to immunotherapy. However, results show that the immune response at the time of diagnosis and upon treatment should encompass immune cells in both the TME and the PB.

## 4. Target Antigen Expression in Pediatric AML

For antigen-directed immunotherapies such as CAR T-cells and bispecific antibodies, the expression of the target antigen on AML blasts is crucial. An ideal target should be strongly expressed on the surface of AML blasts but preferably not or lowly expressed on normal cells. A recent review has summarized the current knowledge on different classes of target antigens in AML, and we refer to that article for a broad overview of this topic [87].

The identification of target antigens has been challenging in both pediatric and adult AML. In addition, target antigen selection for pediatric AML has been largely based on studies from adult patients due to a lack of studies in the pediatric population [88]. Of interest, a recent study by Willier et al. examined immunotherapy targets for pediatric AML (*n* = 36) using RNA-sequencing and flow cytometry and identified distinct expression patterns data in comparison to what has been reported in adult AML [89]. In particular, while CD123 has been shown to be broadly expressed in adult AML, it was lowly expressed in pediatric AML samples. In contrast, a large study that compared CD123 expression between pediatric and adult AML (*n* = 139 and *n* = 316, respectively) reported no differences in expression levels [90].

Moreover, Willier et al. identified CD33 and CLEC12A (CLL1) as a promising combination of targets for immunotherapy covering about 60% of pediatric AML patients [89]. Dual targeting in cancer immunotherapy is a relatively new treatment modality that could be of great interest to reduce off-leukemia effects since the effector cell is only activated in case the target cell expresses both antigens [91,92]. Interestingly, a clinical phase I study is currently ongoing for children and adult AML patients for treatment with CD33-CLEC12A CAR T-cells followed by allo-SCT (ClinicalTrials.gov Identifier: NCT03795779). Preliminary results indicate that this therapy regimen was able to induce remission in a six-year-old girl with secondary AML [93]. Altogether, these findings illustrate the need to uncover immunotarget expression levels prior to clinical application of antigen-directed immunotherapy.

## 5. Immunotherapy Trials in Pediatric AML

**Figure 2:**
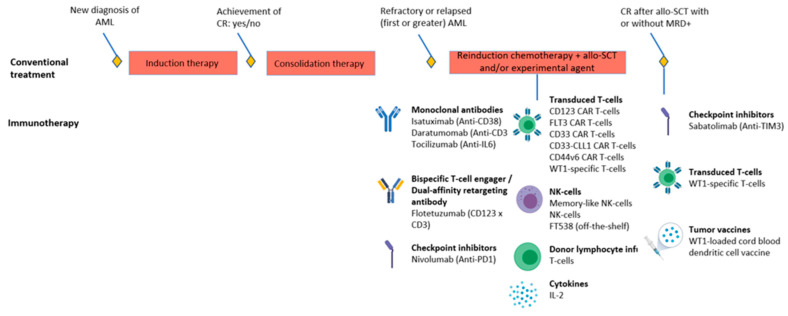
Overview of currently ongoing or planned immunotherapy trials for pediatric AML. Concomitant therapies are presented in Table 2. AML: acute myeloid leukemia; allo-SCT: allogeneic stem cell transplantation; CAR: chimeric antigen receptor; CR: complete remission; PD1: programmed death-1; MRD: minimal residual disease; NK: natural killer; TIM3: T-cell and immunoglobulin and mucin-domain containing-3; WT1; wilms tumor-1.

A complete overview of currently ongoing and recruiting clinical immunotherapy trials for adult AML is presented elsewhere, whereas we focus on immunotherapy trials for pediatric AML in this review [18,19,87] (Figure 2; Table 2). Importantly, we did not focus on trials that aimed to improve the outcomes of allo-SCT by incorporating T- or NK-lymphocytes before or soon after the transplant since this is outside the scope of this review. Except for three studies that focus on the setting of complete remission after allo-SCT, clinical immunotherapy trials for pediatric AML examine relapsed or refractory patients. The overview in Table 2 illustrates the lack of studies that employ the use of ICIs for pediatric AML, which is understandable given the limited success of ICIs as monotherapy in adult AML [18,19]. In pediatric AML, 13 out of 18 immunotherapy trials evaluate the effect of antigen-directed immunotherapies (including unconjugated-, bispecific antibodies, and transduced T-cells). Importantly, only 5 of those 13 trials assess the expression of the target antigen before enrollment. Consequently, this may lead to a lack of response in patients with low or no expression of, for instance, CD123 on AML blasts. Moreover, only 4 out of 18 studies have included immune characterization as part of the trial. As characterization of the immune response in the TME and PB has shown to be informative of why patients respond to immunotherapy, the ability to answer this question will likely be limited. Therefore, we advocate the use of immune profiling efforts in future immunotherapy trials.

## 6. The Way Forward in Pediatric AML: The Need for Immune Profiling

Immune profiling is the overarching term used for quantification and/or characterization of the immune response. Studies in adults with cancer showed that immune profiling data have substantial predictive value for immunotherapy and conventional treatments, illustrated by the development of the Immunoscore^®^ for colorectal cancer and the tumor inflammation score for multiple cancer types [23,24,25,94,95]. However, immune profiling data are scarce in pediatric solid tumors, as reviewed elsewhere, and in pediatric AML, as illustrated in this review [96]. Therefore, as physicians and scientists in the field of pediatric AML, we recognize the urgent need to initiate major immune characterization efforts. Meanwhile, the Society for Immunotherapy of Cancer’s (SITC) has published a resource document on the use of immune profiling for different purposes [97]. In this resource document, the authors describe the discovery of biomarkers that are predictive of both response to immunotherapy and immune-related adverse effects. Another resource document is also available specifically for hematological malignancies [98]. However, these resource documents suggest a multitude of immune profiling methods and there is no consensus on which method(s) should guide the selection of the most beneficial immunotherapy approach for a given (pediatric) AML patient. However, we anticipate that both quantifying and characterizing the systemic and localized immune responses in combination with measuring target antigen expression is most likely to be informative of immunotherapy success.

Accordingly, flow or mass cytometry approaches on bone marrow aspirates and PB or immunohistochemistry on bone biopsies could be used to assess and characterize the immune infiltration of specific immune cell subsets. Importantly, these analyses should include functional and exhaustion markers, such as PD-1 and CTLA4, to identify dysfunctional or exhausted immune cells that may benefit from ICIs. For this purpose, single-cell RNA sequencing could be used to dissect the heterogeneity and dynamics of immune cell subsets in detail [28]. Furthermore, immune cells should be assessed functionally using, for instance, T-cell killing or proliferation assays. In addition, RNA-sequencing data can be used to infer the immune cell abundance utilizing deconvolution algorithms that are validated for AML. Furthermore, TCR and BCR analysis is suitable for monitoring the clonality and the presence of antigen-specific T-cells over the treatment course [48]. Besides, these analyses may provide AML-specific TCRs which may consequently be used to generate genetically engineered autologous or allogeneic T-cells as a novel therapy. Lastly, antigen expression on AML blasts should be assessed by flow cytometry. We envision that after collection and integration of these immune profiling data, generated hypotheses can then be tested in preclinical models with primary samples, to further personalize and optimize the design of clinical immunotherapy trials.

## 7. Discussions

The available evidence about the immune response in the TME and the PB is limited in pediatric AML. Nonetheless, current data suggest that the T- and NK-cell abundance in pediatric AML is comparable to adult AML at diagnosis [29,45]. As a result, we expect that at least a subset of children with AML may also benefit from immunotherapy in the future, as seen in adult AML. However, data regarding the immune cell phenotype at diagnosis, and both the abundance and phenotype during and after treatment, and in the relapsed and refractory setting are still limited for both pediatric and adult AML. Consequently, it is unknown whether biological and/or treatment-related factors such as differences in the underlying pathophysiology and maturation of the immune system cause differences in immune cell parameters between adults and children with AML, or to what extent these differences affect the probability of response to immunotherapy in pediatric AML in comparison to adult AML [38,75,99]. For now, the current data indicate that results from immunotherapy trials in adult AML cannot simply be extrapolated to pediatric AML.

As the level of immune infiltration was shown to be inversely associated with the leukemic burden, there is an increased interest in the use of immunotherapy for the eradication of MRD [56,57,58,59]. Although there is hardly any data on the composition of immune cells in the TME during and after therapy in pediatric AML, results derived from adult AML patients reveal that the use of immunotherapy for the eradication of MRD holds great potential and should be explored further [59]. Moreover, combinations of immunotherapy with other agents such as HMAs and conventional chemotherapy have shown promising results in adult AML and other cancer subtypes [68,69,70,71,72]. These and other innovative approaches will support the transition from the mainly chemotherapy-based treatment in pediatric AML towards a more individualized treatment regimen with increased efficacy and reduced side effects.

## 8. Conclusions

In conclusion, to allow children with AML to benefit from immunotherapy, major immune profiling efforts are needed. These efforts should focus on the systemic and TME immune profile during conventional therapy, as well as in clinical immunotherapy trials. Moreover, future immunotherapy trials should enrich for potential responders to prevent trials with unnecessarily low response rates in a disease with a limited number of patients. These and immunological data-guided preclinical efforts will improve our understanding of why children with AML develop a certain immune profile and which patients are likely to respond to immunotherapy. Importantly, in a rare disease such as pediatric AML, international collaboration will be key to expedite the development and implementation of immunotherapy into patient care.

## Figures and Tables

**Table 1:** Classes of immunotherapy drugs that are studied in AML, mechanisms of action and examples.

Class of Immunotherapy	Main Mechanism of Action	Example for AML	Corresponding Phase(s) of Clinical Trials for Adult AML *
Immune checkpoint inhibitors	Bind to immune checkpoints or their ligands and block their immunosuppressive signal [9]	Nivolumab (anti-PD1) Durvalumab (anti-PDL1)	I–II I–II
Unconjugated antibodies	Bind to tumor specific- or associated antigens and consequently facilitate recruitment of immune effector cells [10]	Daratumumab (anti-CD38)	I–II
Bispecific antibodies	Redirect T- or NK-cells to tumor-specific or -associated antigens [11]	Flotetuzumab (CD3 × CD123) AMG330 (CD3 × CD33)	I–II I
Adoptive cell therapy	Ex vivo transduced or expanded tumor-specific or -associated T- or NK-cells for direct lysis of tumor cells [9]	CAR T-cells directed at CD33 or CD123	I–II I–III
Cytokines and other soluble immune-modulating factors	Growth and activation of T- and NK-cells [9]	IL-2	IV
Vaccines	Increase presentation of tumor specific- or associated antigens [9]	DCP-001 (dendritic cell vaccine)	I–II
Oncolytic viruses	Viral oncolysis of cancer cells and consequent induction of anti-tumor immunity [12]	Vesicular stomatitis virus	I ^†^

AML: acute myeloid leukemia; CAR: chimeric antigen receptor; IL: interleukin; NK: natural killer; PD: programmed death; PDL1: PD ligand 1. * Ongoing trials for pediatric AML are presented in Table 2. ^†^ This trial is currently suspended (clinical hold). More information can be found on www.clinicaltrials.gov (accessed on 15 August 2021).

**Table 2:** Overview of currently ongoing or planned immunotherapy trials for pediatric AML.

Disease Stage	Phase	Drug & Target	Concomitant Therapy	Indication	Ages	Patients	Measures Target Antigen Expression before Enrollment	Performs Immune Characterization	NCT/EUDRACT & Acronym If Applicable	Expected Completion
Relapse/Refractory	II	Isatuximab Anti-CD38	Chemotherapy	R/R	<18 y	96	No	No	NCT03860844 (ISAKIDS)	2022
Relapse/Refractory	II	Memory-like NK-cells	Chemotherapy	R/R	1–21 y	48	NA	No	NCT04354025	2026
Relapse/Refractory	I/II	Nivolumab Anti-PD1	Azacytidine	R/R	1–30 y	26	No	No	NCT03825367	2024
Relapse/Refractory	I/II	Memory-like NK-cells and DLI	Chemotherapy	Relapse after allo-SCT	>0 y	90 (also adults)	NA	No	NCT03068819	2026
Relapse/Refractory	I	Memory-like NK-cells and IL-2	Chemotherapy	R/R	2–17 y	Unclear for pediatric cohort	NA	No	NCT01898793	2028
Relapse/Refractory	I	NK-cells	Chemotherapy	R/R	1–30 y	10 (also adults)	NA	Yes	NCT04327037	2021
Relapse/Refractory	I	CD123 CAR T-cells	Chemotherapy	R/R	>11 y	42 (also adults)	Yes	No	NCT02159495	2021
Relapse/Refractory	I	CD123 CAR T-cells	Chemotherapy	R/R	1–29 y	12	No	No	NCT04678336	2036
Relapse/Refractory	I	CD123 CAR T-cells	Chemotherapy	R/R	<22 y	32	No	Yes	NCT04318678 (CATCHAML)	2025
Relapse/Refractory	I	FLT3 CAR T-cells	-	R/R	>11 y	40 (also adults)	Yes	No	NCT03904069	2029
Relapse/Refractory	I/II	CD33 CAR T-cells	-	R/R	1–35 y	34 (also adults)	Yes	No	NCT03971799	2039
Relapse/Refractory	I	CD33-CLL1 CAR T-cells	-	HR disease	All	20 (also adults)	No	No	NCT03795779	2022
Relapse/Refractory	I/II	CD44v6CAR T-cells	Chemotherapy	R/R	1–17 y for pediatric cohort	58 (also adults)	Yes	No	NCT04097301	2023
Relapse/Refractory	I	Flotetuzumab CD123 × CD3 BiTE	Chemotherapy	R/R	<21 y	47	No	Yes	NCT04158739 (ADVL1812)	2021
Relapse/Refractory	I	Daratumomab Anti-CD38 & FT538 (NK-cell product)	Chemotherapy	R/R after 2 lines of therapy	>11 y	50 (also adults)	Yes	No	NCT04714372	2025
Relapse and post-SCT	I/II	WT1-sensitized allogeneic T-cells and IL-2	Relapse-arm: chemotherapy Post-SCT arm: -	Relapsed after SCT or high risk of relapse post-SCT	Unclear, but weight should be at least 15 kg	45 (also adults)	No	No	NCT01640301	2029
Post-SCT	I/II	Sabatolimab Anti-TIM3	With/without Azacytidine	Post-SCT with CR but MRD+	12–99 y	59 (also adults)	No	No	NCT04623216	2026
Post-SCT	I/II	WT1-loaded cord blood dendritic cell vaccine	-	Post-SCT with CR	0–18 y	54	Yes	Yes	2015-000827-94 (U-DANCE)	Unknown

Allo-SCT: allogeneic stem cell transplantation; BiTE: bispecific T-cell engager, CAR: chimeric antigen receptor; CR: complete remission; DLI: donor lymphocyte infusion; EUDRACT: European drug regulatory authority’s clinical trial; HR: high risk; IL: interleukin; MRD: minimal residual disease; NA: not applicable; NCT: national clinical trial; NK: natural killer; R/R: relapse/refractory disease.

## References

[1] Döhner H., Weisdorf D.J., Bloomfield C.D. (2015). Acute Myeloid Leukemia. N. Engl. J. Med..

[2] Zwaan C.M., Kolb E.A., Reinhardt D., Abrahamsson J., Adachi S., Aplenc R., De Bont E.S., De Moerloose B., Dworzak M., Gibson B.E. (2015). Collaborative Efforts Driving Progress in Pediatric Acute Myeloid Leukemia. J. Clin. Oncol..

[3] Reedijk A.M.J., Klein K., Coebergh J.W.W., Kremer L.C., Dinmohamed A.G., de Haas V., Versluijs A.B., Ossenkoppele G.J., Beverloo H.B., Pieters R. (2019). Improved survival for children and young adolescents with acute myeloid leukemia: A Dutch study on incidence, survival and mortality. Leukemia.

[4] Khaldoyanidi S., Nagorsen D., Stein A., Ossenkoppele G., Subklewe M. (2021). Immune Biology of Acute Myeloid Leukemia: Implications for Immunotherapy. J. Clin. Oncol..

[5] Sendker S., Reinhardt D., Niktoreh N. (2021). Redirecting the Immune Microenvironment in Acute Myeloid Leukemia. Cancers.

[6] Majzner R.G., Heitzeneder S., Mackall C.L. (2017). Harnessing the Immunotherapy Revolution for the Treatment of Childhood Cancers. Cancer Cell.

[7] Orti G., Barba P., Fox L., Salamero O., Bosch F., Valcarcel D. (2017). Donor lymphocyte infusions in AML and MDS: Enhancing the graft-versus-leukemia effect. Exp. Hematol..

[8] Sweeney C., Vyas P. (2019). The Graft-Versus-Leukemia Effect in AML. Front. Oncol..

[9] Disis M.L. (2014). Mechanism of action of immunotherapy. Semin. Oncol..

[10] Ward R.L., Hawkins N.J., Smith G.M. (1997). Unconjugated antibodies for cancer therapy: Lessons from the clinic. Cancer Treat. Rev..

[11] Labrijn A.F., Janmaat M.L., Reichert J.M., Parren P.W.H.I. (2019). Bispecific antibodies: A mechanistic review of the pipeline. Nat. Rev. Drug Discov..

[12] Innao V., Rizzo V., Allegra A.G., Musolino C., Allegra A. (2020). Oncolytic Viruses and Hematological Malignancies: A New Class of Immunotherapy Drugs. Curr. Oncol..

[13] Kantarjian H., Stein A., Gökbuget N., Fielding A.K., Schuh A.C., Ribera J.M., Wei A., Dombret H., Foà R., Bassan R. (2017). Blinatumomab versus Chemotherapy for Advanced Acute Lymphoblastic Leukemia. N. Engl. J. Med..

[14] Blaeschke F., Stenger D., Kaeuferle T., Willier S., Lotfi R., Kaiser A.D., Assenmacher M., Döring M., Feucht J., Feuchtinger T. (2018). Induction of a central memory and stem cell memory phenotype in functionally active CD4^+^ and CD8^+^ CAR T cells produced in an automated good manufacturing practice system for the treatment of CD19^+^ acute lymphoblastic leukemia. Cancer Immunol. Immunother..

[15] Stenger D., Stief T.A., Kaeuferle T., Willier S., Rataj F., Schober K., Vick B., Lotfi R., Wagner B., Grünewald T.G.P. (2020). Endogenous TCR promotes in vivo persistence of CD19-CAR-T cells compared to a CRISPR/Cas9-mediated TCR knockout CAR. Blood.

[16] Eggermont A.M., Maio M., Robert C. (2015). Immune checkpoint inhibitors in melanoma provide the cornerstones for curative therapies. Semin. Oncol..

[17] Ribas A., Wolchok J.D. (2018). Cancer immunotherapy using checkpoint blockade. Science.

[18] Stahl M., Goldberg A.D. (2019). Immune Checkpoint Inhibitors in Acute Myeloid Leukemia: Novel Combinations and Therapeutic Targets. Curr. Oncol. Rep..

[19] Bewersdorf J.P., Zeidan A.M. (2020). Randomized trials with checkpoint inhibitors in acute myeloid leukaemia and myelodysplastic syndromes: What have we learned so far and where are we heading?. Best Pract. Res. Clin. Haematol..

[20] Isidori A., Daver N., Curti A. (2021). Editorial: The Biological Landscape of Immunotherapy in AML. Front. Oncol..

[21] Isidori A., Cerchione C., Daver N., DiNardo C., Garcia-Manero G., Konopleva M., Jabbour E., Ravandi F., Kadia T., Burguera A.F. (2021). Immunotherapy in Acute Myeloid Leukemia: Where We Stand. Front. Oncol..

[22] Binnewies M., Roberts E.W., Kersten K., Chan V., Fearon D.F., Merad M., Coussens L.M., Gabrilovich D.I., Ostrand-Rosenberg S., Hedrick C.C. (2018). Understanding the tumor immune microenvironment (TIME) for effective therapy. Nat. Med..

[23] Bruni D., Angell H.K., Galon J. (2020). The immune contexture and Immunoscore in cancer prognosis and therapeutic efficacy. Nat. Rev. Cancer.

[24] Galon J., Bruni D. (2019). Approaches to treat immune hot, altered and cold tumours with combination immunotherapies. Nat. Rev. Drug Discov..

[25] Bagaev A., Kotlov N., Nomie K., Svekolkin V., Gafurov A., Isaeva O., Osokin N., Kozlov I., Frenkel F., Gancharova O. (2021). Conserved pan-cancer microenvironment subtypes predict response to immunotherapy. Cancer Cell.

[26] Ulloa-Montoya F., Louahed J., Dizier B., Gruselle O., Spiessens B., Lehmann F.F., Suciu S., Kruit W.H., Eggermont A.M., Vansteenkiste J. (2013). Predictive gene signature in MAGE-A3 antigen-specific cancer immunotherapy. J. Clin. Oncol..

[27] Lauss M., Donia M., Harbst K., Andersen R., Mitra S., Rosengren F., Salim M., Vallon-Christersson J., Törngren T., Kvist A. (2017). Mutational and putative neoantigen load predict clinical benefit of adoptive T cell therapy in melanoma. Nat. Commun..

[28] Ren X., Zhang L., Zhang Y., Li Z., Siemers N., Zhang Z. (2021). Insights Gained from Single-Cell Analysis of Immune Cells in the Tumor Microenvironment. Annu. Rev. Immunol..

[29] Vadakekolathu J., Minden M.D., Hood T., Church S.E., Reeder S., Altmann H., Sullivan A.H., Viboch E.J., Patel T., Ibrahimova N. (2020). Immune landscapes predict chemotherapy resistance and immunotherapy response in acute myeloid leukemia. Sci. Transl. Med..

[30] Uy G.L., Aldoss I., Foster M.C., Sayre P.H., Wieduwilt M.J., Advani A.S., Godwin J.E., Arellano M.L., Sweet K.L., Emadi A. (2021). Flotetuzumab as salvage immunotherapy for refractory acute myeloid leukemia. Blood.

[31] Daver N., Garcia-Manero G., Basu S., Boddu P.C., Alfayez M., Cortes J.E., Konopleva M., Ravandi-Kashani F., Jabbour E., Kadia T. (2019). Efficacy, Safety, and Biomarkers of Response to Azacitidine and Nivolumab in Relapsed/Refractory Acute Myeloid Leukemia: A Nonrandomized, Open-Label, Phase II Study. Cancer Discov..

[32] Ragoonanan D., Khazal S.J., Abdel-Azim H., McCall D., Cuglievan B., Tambaro F.P., Ahmad A.H., Rowan C.M., Gutierrez C., Schadler K. (2021). Diagnosis, grading and management of toxicities from immunotherapies in children, adolescents and young adults with cancer. Nat. Rev. Clin. Oncol..

[33] Adam T., Becker T.M., Chua W., Bray V., Roberts T.L. (2021). The Multiple Potential Biomarkers for Predicting Immunotherapy Response-Finding the Needle in the Haystack. Cancers.

[34] Lamble A.J., Tasian S.K. (2019). Opportunities for immunotherapy in childhood acute myeloid leukemia. Blood Adv..

[35] Davidson-Moncada J., Viboch E., Church S.E., Warren S.E., Rutella S. (2018). Dissecting the Immune Landscape of Acute Myeloid Leukemia. Biomedicines.

[36] Lamble A.J., Lind E.F. (2018). Targeting the Immune Microenvironment in Acute Myeloid Leukemia: A Focus on T Cell Immunity. Front. Oncol..

[37] Swatler J., Turos-Korgul L., Kozlowska E., Piwocka K. (2021). Immunosuppressive Cell Subsets and Factors in Myeloid Leukemias. Cancers.

[38] Bolouri H., Farrar J.E., Triche T., Ries R.E., Lim E.L., Alonzo T.A., Ma Y., Moore R., Mungall A.J., Marra M.A. (2018). The molecular landscape of pediatric acute myeloid leukemia reveals recurrent structural alterations and age-specific mutational interactions. Nat. Med..

[39] Fridman W.H., Pagès F., Sautès-Fridman C., Galon J. (2012). The immune contexture in human tumours: Impact on clinical outcome. Nat. Rev. Cancer.

[40] Fridman W.H., Zitvogel L., Sautès-Fridman C., Kroemer G. (2017). The immune contexture in cancer prognosis and treatment. Nat. Rev. Clin. Oncol..

[41] Pagès F., Galon J., Dieu-Nosjean M.C., Tartour E., Sautès-Fridman C., Fridman W.H. (2010). Immune infiltration in human tumors: A prognostic factor that should not be ignored. Oncogene.

[42] Lamble A.J., Kosaka Y., Laderas T., Maffit A., Kaempf A., Brady L.K., Wang W., Long N., Saultz J.N., Mori M. (2020). Reversible suppression of T cell function in the bone marrow microenvironment of acute myeloid leukemia. Proc. Natl. Acad. Sci. USA.

[43] Brück O., Dufva O., Hohtari H., Blom S., Turkki R., Ilander M., Kovanen P., Pallaud C., Ramos P.M., Lähteenmäki H. (2020). Immune profiles in acute myeloid leukemia bone marrow associate with patient age, T-cell receptor clonality, and survival. Blood Adv..

[44] Wang Y., Cai Y.Y., Herold T., Nie R.C., Zhang Y., Gale R.P., Metzeler K.H., Zeng Y., Wang S.Q., Pan X.Y. (2021). An Immune Risk Score Predicts Survival of Patients with Acute Myeloid Leukemia Receiving Chemotherapy. Clin. Cancer Res..

[45] Dufva O., Pölönen P., Brück O., Keränen M.A.I., Klievink J., Mehtonen J., Huuhtanen J., Kumar A., Malani D., Siitonen S. (2020). Immunogenomic Landscape of Hematological Malignancies. Cancer Cell.

[46] Koenig K.L., Sahasrabudhe K.D., Sigmund A.M., Bhatnagar B. (2020). AML with Myelodysplasia-Related Changes: Development, Challenges, and Treatment Advances. Genes.

[47] Bailur J.K., McCachren S.S., Pendleton K., Vasquez J.C., Lim H.S., Duffy A., Doxie D.B., Kaushal A., Foster C., DeRyckere D. (2020). Risk-associated alterations in marrow T cells in pediatric leukemia. JCI Insight.

[48] Gohil S.H., Iorgulescu J.B., Braun D.A., Keskin D.B., Livak K.J. (2021). Applying high-dimensional single-cell technologies to the analysis of cancer immunotherapy. Nat. Rev. Clin. Oncol..

[49] Tumeh P.C., Harview C.L., Yearley J.H., Shintaku I.P., Taylor E.J., Robert L., Chmielowski B., Spasic M., Henry G., Ciobanu V. (2014). PD-1 blockade induces responses by inhibiting adaptive immune resistance. Nature.

[50] Simoni Y., Becht E., Fehlings M., Loh C.Y., Koo S.L., Teng K.W.W., Yeong J.P.S., Nahar R., Zhang T., Kared H. (2018). Bystander CD8^+^ T cells are abundant and phenotypically distinct in human tumour infiltrates. Nature.

[51] Zhang J., Hu X., Wang J., Sahu A.D., Cohen D., Song L., Ouyang Z., Fan J., Wang B., Fu J. (2019). Immune receptor repertoires in pediatric and adult acute myeloid leukemia. Genome Med..

[52] Williams P., Basu S., Garcia-Manero G., Hourigan C.S., Oetjen K.A., Cortes J.E., Ravandi F., Jabbour E.J., Al-Hamal Z., Konopleva M. (2019). The distribution of T-cell subsets and the expression of immune checkpoint receptors and ligands in patients with newly diagnosed and relapsed acute myeloid leukemia. Cancer.

[53] Noviello M., Manfredi F., Ruggiero E., Perini T., Oliveira G., Cortesi F., De Simone P., Toffalori C., Gambacorta V., Greco R. (2019). Bone marrow central memory and memory stem T-cell exhaustion in AML patients relapsing after HSCT. Nat. Commun..

[54] Döhner H., Wei A.H., Löwenberg B. (2021). Towards precision medicine for AML. Nat. Rev. Clin. Oncol..

[55] Michelozzi I.M., Kirtsios E., Giustacchini A. (2021). Driving CAR T Stem Cell Targeting in Acute Myeloid Leukemia: The Roads to Success. Cancers.

[56] Topp M.S., Kufer P., Gökbuget N., Goebeler M., Klinger M., Neumann S., Horst H.A., Raff T., Viardot A., Schmid M. (2011). Targeted therapy with the T-cell-engaging antibody blinatumomab of chemotherapy-refractory minimal residual disease in B-lineage acute lymphoblastic leukemia patients results in high response rate and prolonged leukemia-free survival. J. Clin. Oncol..

[57] Lussana F., Gritti G., Rambaldi A. (2021). Immunotherapy of Acute Lymphoblastic Leukemia and Lymphoma With T Cell-Redirected Bispecific Antibodies. J. Clin. Oncol..

[58] Saudemont A., Quesnel B. (2004). In a model of tumor dormancy, long-term persistent leukemic cells have increased B7-H1 and B7.1 expression and resist CTL-mediated lysis. Blood.

[59] Kadia T.M., Cortes J.E., Ghorab A., Ravandi F., Jabbour E., Daver N.G., Alvarado Y., Ohanian M., Konopleva M., Kantarjian H.M. (2018). Nivolumb (Nivo) maintenance (maint) in high-risk (HR) acute myeloid leukemia (AML) patients. J. Clin. Oncol..

[60] Petit A., Ducassou S., Leblanc T., Pasquet M., Rousseau A., Ragu C., Cachanado M., Nelken B., Bertrand Y., Michel G. (2018). Maintenance Therapy With Interleukin-2 for Childhood AML: Results of ELAM02 Phase III Randomized Trial. Hemasphere.

[61] Nguyen R., Wu H., Pounds S., Inaba H., Ribeiro R.C., Cullins D., Rooney B., Bell T., Lacayo N.J., Heym K. (2019). A phase II clinical trial of adoptive transfer of haploidentical natural killer cells for consolidation therapy of pediatric acute myeloid leukemia. J. Immunother. Cancer.

[62] Gómez García L.M., Escudero A., Mestre C., Fuster S.J.L., Martínez A.P., Vagace Valero J.M., Vela M., Ruz B., Navarro A., Fernández L. (2021). Phase 2 Clinical Trial of Infusing Haploidentical K562-mb15-41BBL-Activated and Expanded Natural Killer Cells as Consolidation Therapy for Pediatric Acute Myeloblastic Leukemia. Clin. Lymphoma Myeloma Leuk..

[63] Knaus H.A., Berglund S., Hackl H., Blackford A.L., Zeidner J.F., Montiel-Esparza R., Mukhopadhyay R., Vanura K., Blazar B.R., Karp J.E. (2018). Signatures of CD8^+^ T cell dysfunction in AML patients and their reversibility with response to chemotherapy. JCI Insight.

[64] Ocadlikova D., Lecciso M., Isidori A., Loscocco F., Visani G., Amadori S., Cavo M., Curti A. (2019). Chemotherapy-Induced Tumor Cell Death at the Crossroads Between Immunogenicity and Immunotolerance: Focus on Acute Myeloid Leukemia. Front. Oncol..

[65] Kanakry C.G., Hess A.D., Gocke C.D., Thoburn C., Kos F., Meyer C., Briel J., Luznik L., Smith B.D., Levitsky H. (2011). Early lymphocyte recovery after intensive timed sequential chemotherapy for acute myelogenous leukemia: Peripheral oligoclonal expansion of regulatory T cells. Blood.

[66] Yang W., Xu Y. (2013). Clinical significance of Treg cell frequency in acute myeloid leukemia. Int. J. Hematol..

[67] Wang M., Zhang C., Tian T., Zhang T., Wang R., Han F., Zhong C., Hua M., Ma D. (2018). Increased Regulatory T Cells in Peripheral Blood of Acute Myeloid Leukemia Patients Rely on Tumor Necrosis Factor (TNF)-α-TNF Receptor-2 Pathway. Front. Immunol..

[68] Vitale I., Shema E., Loi S., Galluzzi L. (2021). Intratumoral heterogeneity in cancer progression and response to immunotherapy. Nat. Med..

[69] Shaked Y. (2019). The pro-tumorigenic host response to cancer therapies. Nat. Rev. Cancer.

[70] Yan Y., Kumar A.B., Finnes H., Markovic S.N., Park S., Dronca R.S., Dong H. (2018). Combining Immune Checkpoint Inhibitors With Conventional Cancer Therapy. Front. Immunol..

[71] Derer A., Frey B., Fietkau R., Gaipl U.S. (2016). Immune-modulating properties of ionizing radiation: Rationale for the treatment of cancer by combination radiotherapy and immune checkpoint inhibitors. Cancer Immunol. Immunother..

[72] Käsmann L., Eze C., Dantes M., Roengvoraphoj O., Niyazi M., Belka C., Manapov F. (2019). State of clinical research of radiotherapy/chemoradiotherapy and immune checkpoint inhibitor therapy combinations in solid tumours-a German radiation oncology survey. Eur. J. Cancer.

[73] Mendez L.M., Posey R.R., Pandolfi P.P. (2019). The Interplay Between the Genetic and Immune Landscapes of AML: Mechanisms and Implications for Risk Stratification and Therapy. Front. Oncol..

[74] Wellenstein M.D., de Visser K.E. (2018). Cancer-Cell-Intrinsic Mechanisms Shaping the Tumor Immune Landscape. Immunity.

[75] Chaudhury S., O’Connor C., Cañete A., Bittencourt-Silvestre J., Sarrou E., Prendergast Á., Choi J., Johnston P., Wells C.A., Gibson B. (2018). Age-specific biological and molecular profiling distinguishes paediatric from adult acute myeloid leukaemias. Nat. Commun..

[76] Hiam-Galvez K.J., Allen B.M., Spitzer M.H. (2021). Systemic immunity in cancer. Nat. Rev. Cancer.

[77] Yost K.E., Satpathy A.T., Wells D.K., Qi Y., Wang C., Kageyama R., McNamara K.L., Granja J.M., Sarin K.Y., Brown R.A. (2019). Clonal replacement of tumor-specific T cells following PD-1 blockade. Nat. Med..

[78] Wu T.D., Madireddi S., de Almeida P.E., Banchereau R., Chen Y.J., Chitre A.S., Chiang E.Y., Iftikhar H., O’Gorman W.E., Au-Yeung A. (2020). Peripheral T cell expansion predicts tumour infiltration and clinical response. Nature.

[79] Valpione S., Galvani E., Tweedy J., Mundra P.A., Banyard A., Middlehurst P., Barry J., Mills S., Salih Z., Weightman J. (2020). Immune-awakening revealed by peripheral T cell dynamics after one cycle of immunotherapy. Nat. Cancer.

[80] Bansal A.K., Sharawat S.K., Gupta R., Vishnubhatla S., Dhawan D., Bakhshi S. (2020). Regulatory T cells in pediatric AML are associated with disease load and their serial assessment suggests role in leukemogenesis. Am. J. Blood Res..

[81] Admiraal R., van Kesteren C., Jol-van der Zijde C.M., Lankester A.C., Bierings M.B., Egberts T.C., van Tol M.J., Knibbe C.A., Bredius R.G., Boelens J.J. (2015). Association between anti-thymocyte globulin exposure and CD4+ immune reconstitution in paediatric haemopoietic cell transplantation: A multicentre, retrospective pharmacodynamic cohort analysis. Lancet Haematol..

[82] de Koning C., Admiraal R., Nierkens S., Boelens J.J. (2017). Immune reconstitution and outcomes after conditioning with anti-thymocyte-globulin in unrelated cord blood transplantation; the good, the bad, and the ugly. Stem. Cell Investig..

[83] Chiesa R., Gilmour K., Qasim W., Adams S., Worth A.J., Zhan H., Montiel-Equihua C.A., Derniame S., Cale C., Rao K. (2012). Omission of in vivo T-cell depletion promotes rapid expansion of naïve CD4^+^ cord blood lymphocytes and restores adaptive immunity within 2 months after unrelated cord blood transplant. Br. J. Haematol..

[84] Admiraal R., Lindemans C.A., van Kesteren C., Bierings M.B., Versluijs A.B., Nierkens S., Boelens J.J. (2016). Excellent T-cell reconstitution and survival depend on low ATG exposure after pediatric cord blood transplantation. Blood.

[85] Soiffer R.J., Davids M.S., Chen Y.B. (2018). Tyrosine kinase inhibitors and immune checkpoint blockade in allogeneic hematopoietic cell transplantation. Blood.

[86] Wang A.Y., Kline J., Stock W., Kosuri S., Artz A., Larson R.A., Riedell P.A., Bishop M., Liu H. (2020). Unexpected Toxicities When Nivolumab Was Given as Maintenance Therapy following Allogeneic Stem Cell Transplantation. Biol. Blood Marrow Transpl..

[87] Daver N., Alotaibi A.S., Bücklein V., Subklewe M. (2021). T-cell-based immunotherapy of acute myeloid leukemia: Current concepts and future developments. Leukemia.

[88] De Moerloose B. (2021). CAR-T treatment of pediatric AML: A long and winding road. Blood.

[89] Willier S., Rothämel P., Hastreiter M., Wilhelm J., Stenger D., Blaeschke F., Rohlfs M., Kaeuferle T., Schmid I., Albert M.H. (2021). CLEC12A and CD33 coexpression as a preferential target for pediatric AML combinatorial immunotherapy. Blood.

[90] Bras A.E., de Haas V., van Stigt A., Jongen-Lavrencic M., Beverloo H.B., Te Marvelde J.G., Zwaan C.M., van Dongen J.J.M., Leusen J.H.W., van der Velden V.H.J. (2019). CD123 expression levels in 846 acute leukemia patients based on standardized immunophenotyping. Cytom. B Clin. Cytom..

[91] Shah N.N., Maatman T., Hari P., Johnson B. (2019). Multi Targeted CAR-T Cell Therapies for B-Cell Malignancies. Front. Oncol..

[92] Kloss C.C., Condomines M., Cartellieri M., Bachmann M., Sadelain M. (2013). Combinatorial antigen recognition with balanced signaling promotes selective tumor eradication by engineered T cells. Nat. Biotechnol..

[93] Liu F., Cao Y., Pinz K., Ma Y., Wada M., Chen K., Ma G., Shen J., Tse C.O., Su Y. (2018). First-in-human CLL1-CD33 compount CAR T cell therapy induces complete remission in patients with refractory acute myeloid leukemia: Update on phase 1 clinical trial. Blood.

[94] Danaher P., Warren S., Lu R., Samayoa J., Sullivan A., Pekker I., Wallden B., Marincola F.M., Cesano A. (2018). Pan-cancer adaptive immune resistance as defined by the Tumor Inflammation Signature (TIS): Results from The Cancer Genome Atlas (TCGA). J. Immunother. Cancer.

[95] Danaher P., Warren S., Dennis L., D’Amico L., White A., Disis M.L., Geller M.A., Odunsi K., Beechem J., Fling S.P. (2017). Gene expression markers of Tumor Infiltrating Leukocytes. J. Immunother. Cancer.

[96] Terry R.L., Meyran D., Ziegler D.S., Haber M., Ekert P.G., Trapani J.A., Neeson P.J. (2020). Immune profiling of pediatric solid tumors. J. Clin. Investig..

[97] Hu-Lieskovan S., Bhaumik S., Dhodapkar K., Grivel J.J.B., Gupta S., Hanks B.A., Janetzki S., Kleen T.O., Koguchi Y., Lund A.W. (2020). SITC cancer immunotherapy resource document: A compass in the land of biomarker discovery. J. Immunother. Cancer.

[98] Zafeiris D., Vadakekolathu J., Wagner S., Pockley A.G., Ball G.R., Rutella S. (2017). Discovery and application of immune biomarkers for hematological malignancies. Expert Rev. Mol. Diagn..

[99] Simon A.K., Hollander G.A., McMichael A. (2015). Evolution of the immune system in humans from infancy to old age. Proc. Biol. Sci..

